# Blood-brain barrier water exchange and paramagnetic susceptibility alterations during anti-amyloid therapy: preliminary MRI findings

**DOI:** 10.1016/j.tjpad.2025.100256

**Published:** 2025-06-30

**Authors:** Yuto Uchida, Yuya Kano, Hirohito Kan, Keita Sakurai, Hideyasu Morita, Yoshihiro Akagawa, Noriyuki Matsukawa, Kenichi Oishi

**Affiliations:** a Department of Radiology and Radiological Science, Johns Hopkins University School of Medicine, 208 Traylor Building, 720 Rutland Avenue, Baltimore, MD 21205, USA; b Department of Neurology, Nagoya City University Graduate School of Medical Sciences, 1, Kawasumi, Mizuho-cho, Mizuho-ku, Nagoya, Aichi 467-8601, Japan; c Department of Neurology, Toyokawa City Hospital, 23, Noji, Yawata-cho, Toyokawa, Aichi 442-0857, Japan; d Department of Integrated Health Sciences, Nagoya University Graduate School of Medicine, 1-1-20, Daiko-Minami, Higashi-ku, Nagoya, Aichi 461-8673, Japan; e Department of Radiology, National Center for Geriatrics and Gerontology, Morioka-cho, Obu, Aichi 474-8511, Japan; f Department of Radiology, Toyokawa City Hospital, 23, Noji, Yawata-cho, Toyokawa, Aichi 442-0857, Japan; g The Richman Family Precision Medicine Center of Excellence in Alzheimer's Disease, Baltimore, MD 21224, USA

To the Editor,

Anti-amyloid-β (Aβ) monoclonal antibodies, including lecanemab, have demonstrated efficacy in reducing amyloid burden and slowing cognitive decline in early Alzheimer’s disease (AD) [1]. However, their administration is often accompanied by amyloid-related imaging abnormalities (ARIA), such as ARIA-H (hemosiderin deposition) and ARIA-E (edema or effusion), which complicate treatment safety and monitoring [2]. Given the role of the blood-brain barrier (BBB) in Aβ transport and ARIA pathogenesis (Supplementary Figure 1), [3] non-invasive imaging of BBB function and vascular integrity may offer valuable insights for optimizing therapy.

In our prospective, observational study (UMIN000055333), 31 patients with early AD received biweekly intravenous lecanemab. MRI and cognitive assessments were performed monthly over a 3-month period (Supplementary Figure 2). We employed diffusion-prepared pseudo-continuous arterial spin labeling (DP-pCASL) to quantify BBB water exchange rate (*k_w_*), [4] and quantitative susceptibility mapping with source separation (QSM-ARCS) to distinguish paramagnetic (e.g., iron) and diamagnetic (e.g., myelin) tissue components [5].

ARIA-H developed asymptomatically in 3 of 31 participants (10 %), all of whom were *APOE* ε4 carriers (Supplementary Table 1). Notably, each exhibited a transient increase in *k_w_* prior to ARIA-H onset and sustained elevation of paramagnetic susceptibility thereafter (Fig. 1 and Supplementary Figure 3). These dynamic imaging patterns were not observed in participants without ARIA-H. Diamagnetic susceptibility values remained stable across the cohort.

**Fig. 1:**
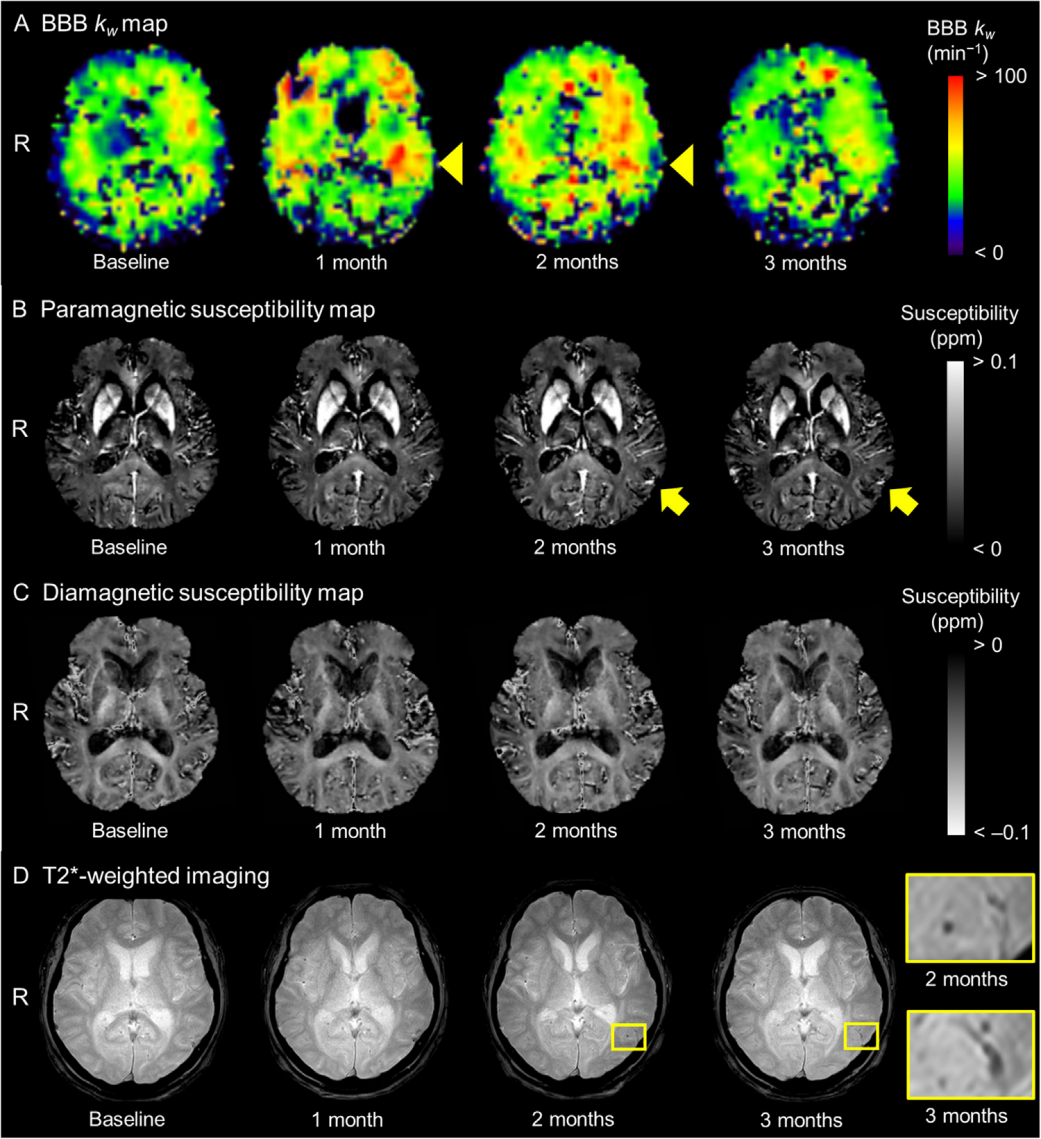
Representative images. Longitudinal changes in representative BBB *k_w_* maps (Fig. 1A) and the corresponding paramagnetic (Fig. 1B) and diamagnetic susceptibility maps (Fig. 1C) are presented from an eligible participant who developed ARIA-H accompanying microbleeds detected on T2*-weighted imaging (Fig. 1D) in the left lateral temporal lobe at 2 months post-treatment of anti-Aβ monoclonal antibodies. Yellow bounding boxes are 10 × magnified to visualize the microbleeds. BBB *k_w_* values transiently increased before the onset of ARIA-H, and this increase was more pronounced in regions exhibiting ARIA-H than in unaffected areas (yellow arrowheads). Paramagnetic susceptibility maps taken after the manifestation of ARIA-H exhibited increases in paramagnetic susceptibility values compared to those without ARIA-H (yellow arrows). Meanwhile, diamagnetic susceptibility values remained relatively stable across the images over time. ARIA-H = ARIA with hemosiderin deposits, BBB = blood-brain barrier, *R* = right.

Linear mixed-effects models (Supplementary Table 2) revealed that Montreal Cognitive Assessment (MoCA) scores were positively correlated with *k_w_* in the frontal (*β* = 0.471; FDR-corrected *P* = 0.019) and medial temporal lobes (*β* = 0.419; FDR-corrected *P* = 0.038), and negatively correlated with paramagnetic susceptibility in the medial (*β* = −0.524; *P* = 0.005) and lateral temporal lobes (*β* = −0.438; *P* = 0.027). These associations suggest that increased BBB permeability may reflect glymphatic activity or vascular health, while elevated paramagnetic susceptibility may mark subclinical vascular damage or iron deposition.

Our findings support the feasibility of using DP-pCASL and QSM-ARCS for real-time monitoring of treatment-related neurovascular changes. The *k_w_* metric may serve as a sensitive early indicator of ARIA risk, and paramagnetic susceptibility may track iron-related pathology. Together, these biomarkers show potential for guiding individualized treatment and safety protocols.

While limited by sample size and follow-up duration, this study provides proof-of-concept for integrating advanced MRI into clinical workflows for AD. Future multicenter trials with larger cohorts are needed to confirm these observations and define clinically actionable thresholds.

In conclusion, advanced MRI biomarkers of BBB function and magnetic susceptibility represent a promising approach for improving safety monitoring, elucidating the mechanisms of ARIA, and personalizing anti-amyloid therapy in patients with AD.

## Consent for publication

All authors read the manuscript and agreed to its publication.

## Ethical approval and consent to participate

The ethics committees for human research of Toyokawa City Hospital-Internal Review Board (TCH-IRB) approved the study design and protocol (TCH-IRB Number: 2024–9–130). Written informed consent was obtained from all participants.

## Funding

This study was supported by the following grants from the Japan Society for the Promotion of Science (KAKENHI): 22K07520 to YU and 23K07107 to HK. YU has received grants from the Alzheimer’s Association and the National Alzheimer’s Coordinating Center (NIAP24–1268927) and the National Institute on Aging (K99AG088363) outside the submitted work in the past 36 months (the payments are made to Johns Hopkins University School of Medicine).

## Data availability statement

The datasets generated and analyzed during the current study are available from the corresponding author upon reasonable request.

## Declaration of generative AI and AI-assisted technologies in the writing process

We have not used any AI at all.

## CRediT authorship contribution statement

**Yuto Uchida:** Conceptualization, Data curation, Formal analysis, Funding acquisition, Investigation, Methodology, Project administration, Visualization, Writing – original draft. **Yuya Kano:** Conceptualization, Data curation, Investigation, Methodology, Visualization, Writing – review & editing. **Hirohito Kan:** Formal analysis, Funding acquisition, Investigation, Methodology, Software, Visualization, Writing – review & editing. **Keita Sakurai:** Investigation, Methodology, Writing – review & editing. **Hideyasu Morita:** Data curation, Investigation, Methodology, Writing – review & editing. **Yoshihiro Akagawa:** Data curation, Investigation, Methodology, Software, Writing – review & editing. **Noriyuki Matsukawa:** Conceptualization, Supervision, Writing – review & editing. **Kenichi Oishi:** Conceptualization, Supervision, Validation, Writing – review & editing.

## Declaration of competing interest

The authors declare the following financial interests/personal relationships which may be considered as potential competing interests:

Yuto Uchida reports financial support was provided by Japan Society for the Promotion of Science. Hirohito Kan reports financial support was provided by Japan Society for the Promotion of Science. Yuto Uchida reports financial support was provided by Alzheimer’s Association. Yuto Uchida reports financial support was provided by National Institute on Aging. If there are other authors, they declare that they have no known competing financial interests or personal relationships that could have appeared to influence the work reported in this paper.

## Appendix Supplementary materials

Image, application 1
