# Supplementary material for: Blood-brain barrier water exchange and paramagnetic susceptibility alterations during anti-amyloid therapy: preliminary MRI findings
Source: J Prev Alzheimers Dis. 2025 Jun 30;12(8):100256. doi: 10.1016/j.tjpad.2025.100256 (PMC12413729; doi:10.1016/j.tjpad.2025.100256)
Supplement: Supplementary file 1 [file mmc1.pdf]

Supplementary Materials

Supplementary Table 1. Clinical characteristics of participants with and without ARIA

Supplementary Table 2. Coefficient estimates in the linear mixed-effects models

Supplementary Figure 1. A schematic hypothesis

Supplementary Figure 2. Graphical study design

Supplementary Figure 3. Longitudinal plots of k_w, paramagnetic, and diamagnetic susceptibility values in all participants

The supplemental materials have been provided by the authors to give readers additional information about their work.

Supplementary Table 1. Clinical characteristics of participants with and without ARIA

**Characteristics**	**Participants without ARIA**	**Participants with ARIA**	***P* value**
Total, No. of participants	28	3	NA
Sex, No. of women (%)	22 (71)	22 (73)	0.625
Age at baseline MRI scan ± SD, years	74.6 ± 4.4	77.6 ± 1.5	0.071
APOE genotypes			
ε2/ε3 (%)	1 (4)	0 (0)	1.000
ε3/ε3 (%)	14 (50)	0 (0)	0.232
ε3/ε4 (%)	9 (32)	2 (67)	0.281
ε4/ε4 (%)	4 (14)	1 (33)	0.422
Neuropsychological assessments at baseline			
Clinical Dementia Rating (Q1, Q3)	0.5 (0.5, 0.5)	0.5 (0.5, 0.5)	0.903
Mini-Mental State Examination	25.1 ± 2.4	23.7 ± 1.2	0.152
Montreal Cognitive Assessment	17.1 ± 3.0	17.3 ± 4.1	0.823
Alzheimer's Disease Assessment Scale	16.1 ± 5.8	18.0 ± 4.4	0.534
Logical Memory I	5.7 ± 1.2	5.9 ± 3.9	0.896
Logical Memory II (Q1, Q3)	0 (0, 2)	0 (0, 1)	0.594
Apathy Rating Scale (Q1, Q3)	12.0 ± 8.7	9.5 ± 4.6	0.238
Geriatric Depression Scale	2.9 ± 2.3	2.3 ± 1.2	0.523
Neuropsychiatric Inventory-Questionnaire (Q1, Q3)	2 (0, 4)	2 (1, 6)	0.912
Zarit Caregiver Burden Interview (Q1, Q3)	1 (0, 2)	1 (0, 4)	0.782
Neuropsychological assessments at 3 months post-treatment			
Clinical Dementia Rating (Q1, Q3)	0.5 (0.5, 0.5)	0.5 (0.5, 0.5)	0.903
Mini-Mental State Examination	24.5 ± 2.1	23.3 ± 0.6	0.341
Montreal Cognitive Assessment	19.0 ± 3.1	17.7 ± 2.5	0.113
Alzheimer's Disease Assessment Scale	14.0 ± 4.7	15.3 ± 2.5	0.255
Logical Memory I	4.7 ± 3.8	5.3 ± 1.6	0.216
Logical Memory II (Q1, Q3)	0 (0, 2)	0 (0, 1)	0.647
Apathy Rating Scale (Q1, Q3)	11.4 ± 6.8	8.4 ± 5.4	0.313
Geriatric Depression Scale	2.9 ± 2.4	1.8 ± 2.5	0.633
Neuropsychiatric Inventory-Questionnaire (Q1, Q3)	3 (2, 6)	2 (1, 6)	0.602

PET, positron emission tomography; Q1, Quartile 1; Q3, Quartile 3; SD, standard deviation.

Supplementary Table 2. Coefficient estimates in the linear mixed-effects models

**Dependent variable:** MoCA score<br>**Independent variables:** BBB k_w value	**Coefficient *β* (95% CI)**	**FDR-corrected *P* value**
Frontal lobe	0.471 (0.116, 0.825)	0.019*
Medial temporal lobe	0.419 (0.071, 0.758)	0.038*
Lateral temporal lobe	0.332 (−0.071, 0.723)	0.081
Parietal lobe	0.263 (−0.120, 0.644)	0.107
Precuneus	0.175 (−0.153, 0.506)	0.198
Posterior cingulate gyrus	0.106 (−0.212, 0.422)	0.502

**Dependent variable:** MoCA score<br>**Independent variables:** Paramagnetic susceptibility value	**Coefficient *β* (95% CI)**	**FDR-corrected *P* value**
Frontal lobe	−0.213 (−0.535, 0.117)	0.156
Medial temporal lobe	−0.524 (−0.893, −0.156)	0.005**
Lateral temporal lobe	−0.438 (−0.772, −0.088)	0.027*
Parietal lobe	−0.281 (−0.640, 0.081)	0.078
Precuneus	−0.162 (−0.451, 0.125)	0.215
Posterior cingulate gyrus	−0.118 (−0.376, 0.143)	0.414

**Dependent variable:** MoCA score<br>**Independent variables:** Diamagnetic susceptibility value	**Coefficient *β* (95% CI)**	**FDR-corrected *P* value**
Frontal lobe	−0.138 (−0.391, 0.120)	0.372
Medial temporal lobe	0.121 (−0.195, 0.437)	0.431
Lateral temporal lobe	0.094 (−0.219, 0.403)	0.516
Parietal lobe	−0.125 (−0.442, 0.193)	0.405
Precuneus	−0.087 (−0.339, 0.164)	0.589
Posterior cingulate gyrus	0.055 (0.310, −0.203)	0.626

Significant differences are denoted as *FDR-corrected *P* value < 0.05 and **FDR-corrected *P* value < 0.01.

Supplementary Figure 1. A schematic hypothesis showing pathophysiological mechanisms of anti-amyloid-β (Aβ) monoclonal antibodies and amyloid-related imaging abnormalities (ARIA). When antibodies bind to Aβ aggregates accumulated in brain parenchyma or vessel walls, solubilized Aβ is excreted from perivascular spaces, leading to a stagnation of vascular permeability (pathomechanism 1). Subsequently, extravasation of erythrocytes and plasma occurs after vascular wall breakdown, contributing to the development of ARIA (pathomechanism 2).

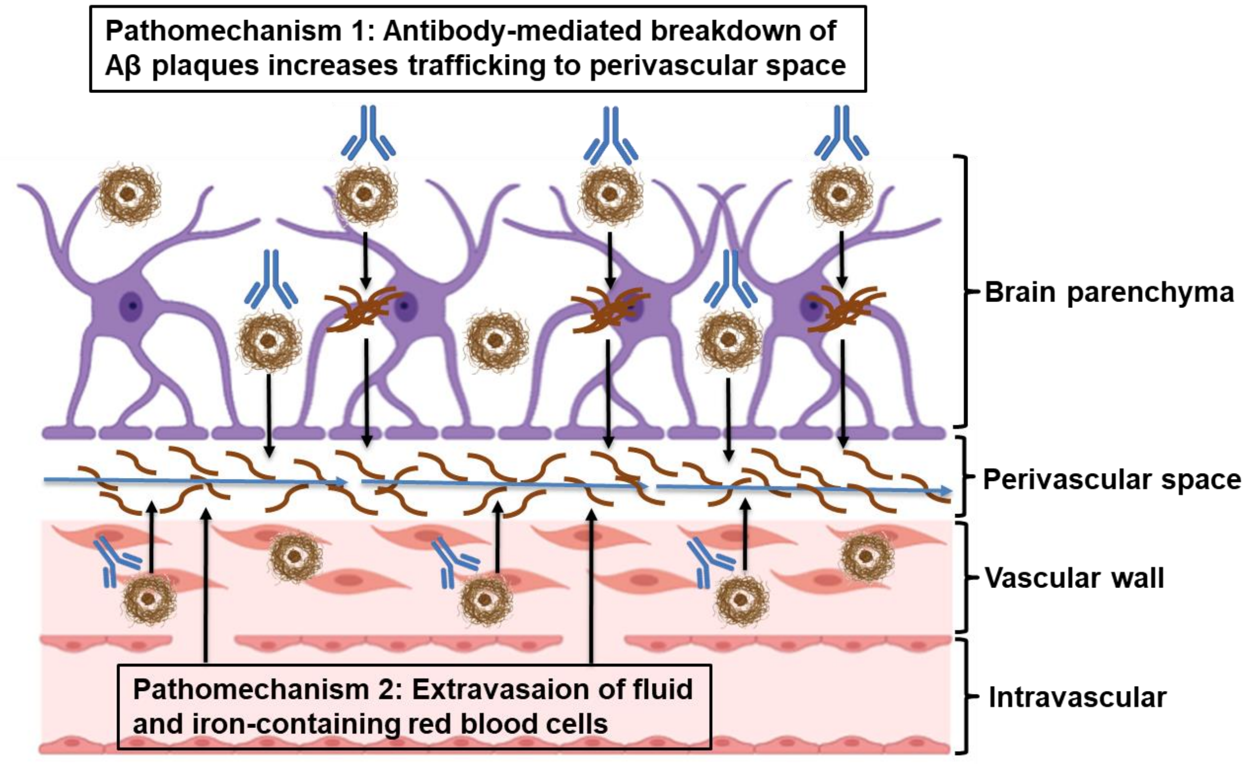

Supplementary Figure 2. Graphical study design. The study participants comprised consecutive patients between April 2024 and March 2025 to whom lecanemab (Leqembi®, Eisai, Tokyo, Japan) was intravenously administered at 10 mg per kilogram of body weight every 2 weeks. The patients who agreed to participate in this study were scanned with MRI sequences (T1WI, T2WI, T2*WI, FLAIR, DWI, MRA, DP pCASL, 3D T1-FFE). The date of the baseline MRI acquisition was within 2 months before initiating infusions of lecanemab. The participants were followed with monthly MRI scans until 3 months after the treatment started (i.e., 1 month, 2 months, and 3 months post-treatment). Neuropsychological assessments (CDR, MMSE, MoCA, ADAS-Cog, WMS-R, NPI, Apathy Scale, GDS, ZCBI) were assessed at the time of the baseline and 3 months post-treatment MRI acquisition.

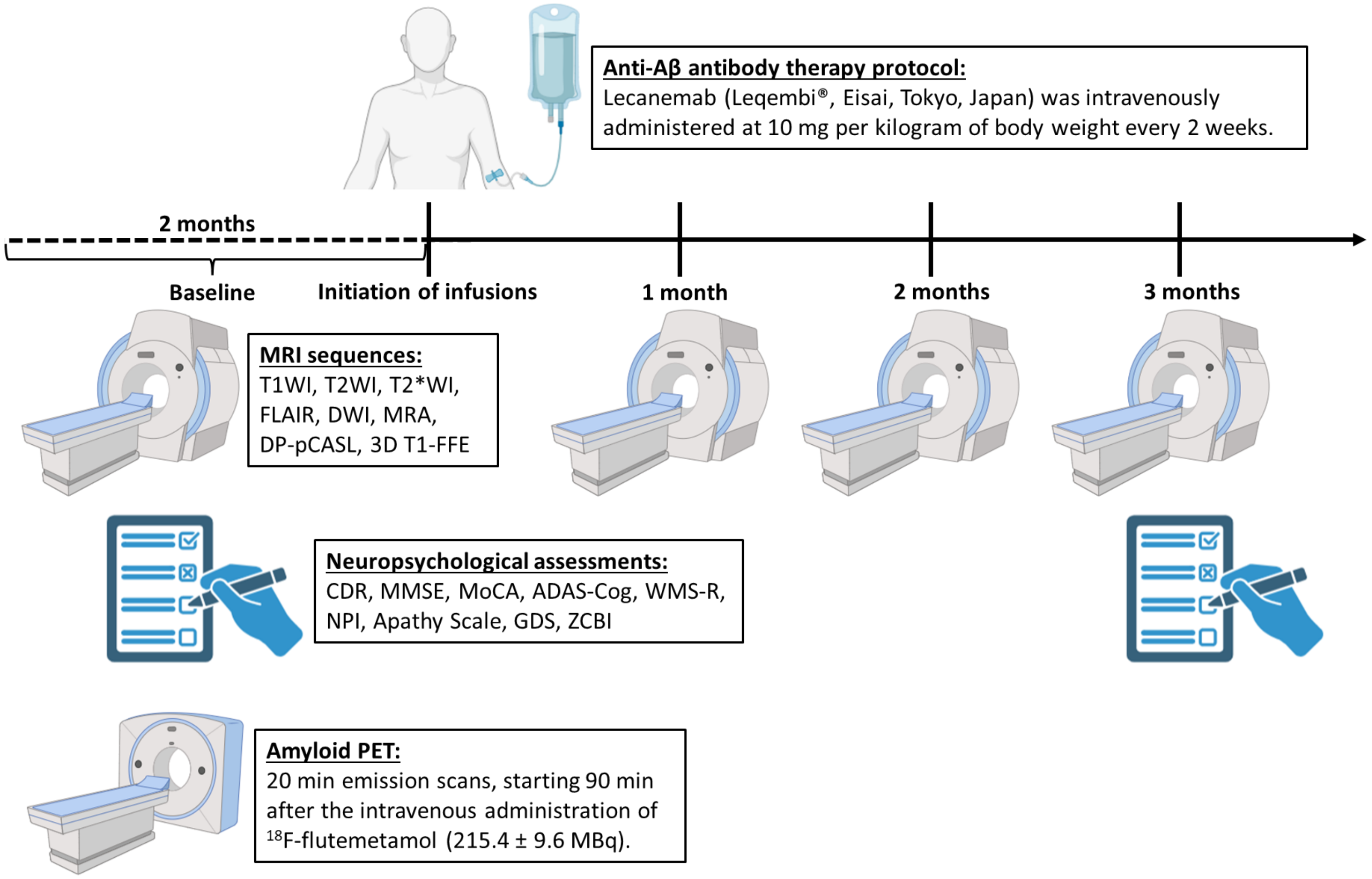

Abbreviations of MRI sequences: T1WI, T1-weighted imaging; T2WI, T2-weighted imaging; T2*WI, T2*-weighted imaging; FLAIR, fluid-attenuated inversion recovery; DWI, diffusion-weighted imaging; MRA, magnetic resonance angiography; DP-pCASL, diffusion-prepared pseudo-continuous arterial spin labeling; 3D T1-FFE, three-dimensional T1-fast field echo.
Abbreviations of neuropsychological assessments: CDR, Clinical Dementia Rating; MMSE, Mini-Mental State Examination; MoCA, Montreal Cognitive Assessment; ADAS-Cog, Alzheimer's Disease Assessment Scale-Cognitive Subscale; WMS-R, Wechsler Memory Scale-Revised; NPI, Neuropsychiatric Inventory; GDS, Geriatric Depression Scale; ZCBI, Zarit Caregiver Burden Interview.

Supplementary Figure 3. Longitudinal plots of k_w, paramagnetic, and diamagnetic susceptibility values in all participants. Longitudinal changes in each quantitative MRI value are plotted as a function of months from the initiation of anti-Aβ monoclonal antibodies in the following predefined regions of relevance to AD pathology: the frontal lobe, medial temporal lobe, lateral temporal lobe, parietal lobe, precuneus, and posterior cingulate gyrus. Red-colored lines are individuals with ARIA, while blue-colored lines are individuals without ARIA. Red arrows indicate the date of ARIA diagnosis and are shown only in the panels demonstrating significant differences. Significant differences are denoted as *FDR-corrected *P* value < 0.05 and **FDR-corrected *P* value < 0.01.

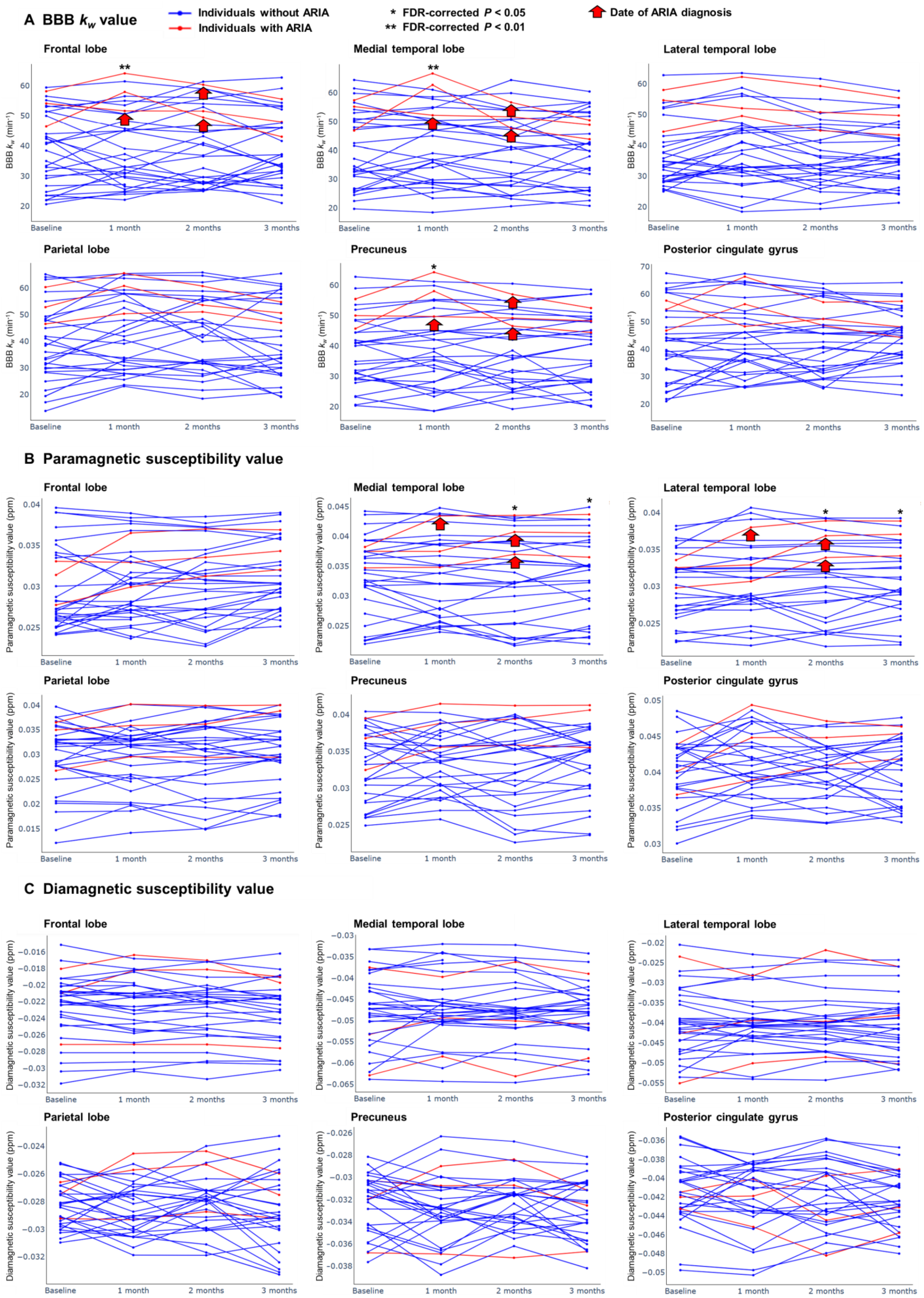
A BBB kw value
Individuals without ARIA
Individuals with ARIA
* FDR-corrected P < 0.05
** FDR-corrected P < 0.01
Date of ARIA diagnosis
Frontal lobe
Medial temporal lobe
Lateral temporal lobe
Parietal lobe
Precuneus
Posterior cingulate gyrus
BBB kw (min-1)
Baseline
1 month
2 months
3 months
B Paramagnetic susceptibility value
Paramagnetic susceptibility value (ppm)
C Diamagnetic susceptibility value
Diamagnetic susceptibility value (ppm)
